# Ultrasensitive Ti_3_C_2_T_X_ MXene/Chitosan Nanocomposite-Based Amperometric Biosensor for Detection of Potential Prostate Cancer Marker in Urine Samples

**DOI:** 10.3390/pr8050580

**Published:** 2020-05-13

**Authors:** Stefania Hroncekova, Tomas Bertok, Michal Hires, Eduard Jane, Lenka Lorencova, Alica Vikartovska, Aisha Tanvir, Peter Kasak, Jan Tkac

**Affiliations:** 1 Institute of Chemistry, Slovak Academy of Sciences, Dubravska cesta 9, Bratislava 845 38, Slovak Republic; 2 Center for Advanced Materials, Qatar University, P. O. BOX 2713, Doha, Qatar

**Keywords:** MXene, nanocomposite, biosensor, sarcosine, sarcosine oxidase, prostate cancer

## Abstract

Two-dimensional layered nanomaterial Ti_3_C_2_T_X_ (a member of the MXene family) was used to immobilise enzyme sarcosine oxidase to fabricate a nanostructured biosensor. The device was applied for detection of sarcosine, a potential prostate cancer biomarker, in urine for the first time. The morphology and structures of MXene have been characterised by atomic force microscopy (AFM) and scanning electron microscopy (SEM). Electrochemical measurements, SEM and AFM analysis revealed that MXene interfaced with chitosan is an excellent support for enzyme immobilisation to fabricate a sensitive biosensor exhibiting a low detection limit of 18 nM and a linear range up to 7.8 µM. The proposed biosensing method also provides a short response time of 2 s and high recovery index of 102.6% for detection of sarcosine spiked into urine sample in a clinically relevant range.

## 1 Introduction

Early stage diagnostics of prostate cancer (PCa) and therapy monitoring of PCa patients is done by analysis of the level of prostate-specific antigen (PSA) in blood. PSA is a glycoprotein released into the blood stream by the prostate and the protein is responsible for seminal fluid liquefaction. Since PSA is organ-specific rather than cancer-specific, the protein is released into the blood stream not only as a result of PCa development and progression, but under other conditions such as prostate inflammation. Hence, PSA cannot be used as a reliable early stage diagnostic PCa biomarker because of the high false-positive/negative results provided [1, 2], with an AUC (Area Under Curve in Receiver Operating Curve) of 0.68 [1]. PCa is one of the most frequently diagnosed cancers, with the second highest mortality rate among men globally [3]. Thus, there is an urgent need to find new sensitive and specific biomarkers for PCa to reduce the number of unnecessary biopsies and to enhance PCa patient survival. One of the alternative PCa biomarkers is sarcosine, which is able to provide significant discrimination between PCa patients and non-cancer individuals, with an AUC of 0.833 [4]. A wide range of (bio)analytical approaches are available for determination of sarcosine, such as a colorimetric [5], enzymatic [6], electrochemical and/or spectrometric [7] and liquid chromatography with tandem mass spectrometry-based approaches [8]. All of these methods have drawbacks such as low sensitivity and/or specificity, time-consuming sample preparation and a need for having skilled persons to operate such instrumentation [9].

Two-dimensional (2D) nanomaterials, namely graphene [10], find numerous applications because of their high surface area and unique electronic/optical properties that differ significantly from their bulk counterparts [11]. Although graphene has attracted more attention than any other 2D nanomaterial so far, a simple interfacial chemistry limits some applications. 2D nanomaterials containing more than one element offer novel possibilities as they provide larger numbers of compositional variables, which can be finely tuned to achieve specific properties [12]. Since their appearance in 2011, 2D transition metal carbides/nitrides, known as MXenes, due to their metallic conductivity, large electrochemically active surface, excellent mechanical properties and water solubility, are extensively used in many applications such as batteries/super-capacitators, sensors and biosensors [13–22].

So far, only a limited number of MXenes-based biosensors have been developed [17]. A pristine or modified Ti_3_C_2_ MXene and haemoglobin-based device was developed for determination of H_2_O_2_ [23] or NaNO_2_ [24]. MXene, with a hydrophilic surface and excellent metallic conductivity, is a prospective interface for effective docking of a wide range of biorecognition molecules for preparation of biosensors. Enzymatic MXene-based electrochemical biosensors have been recently prepared by immobilisation of glucose oxidase [25], acetylcholinesterase [26] and tyrosinase [27] for ultrasensitive and rapid detection of glucose or toxic compounds.

**Figure 1:**
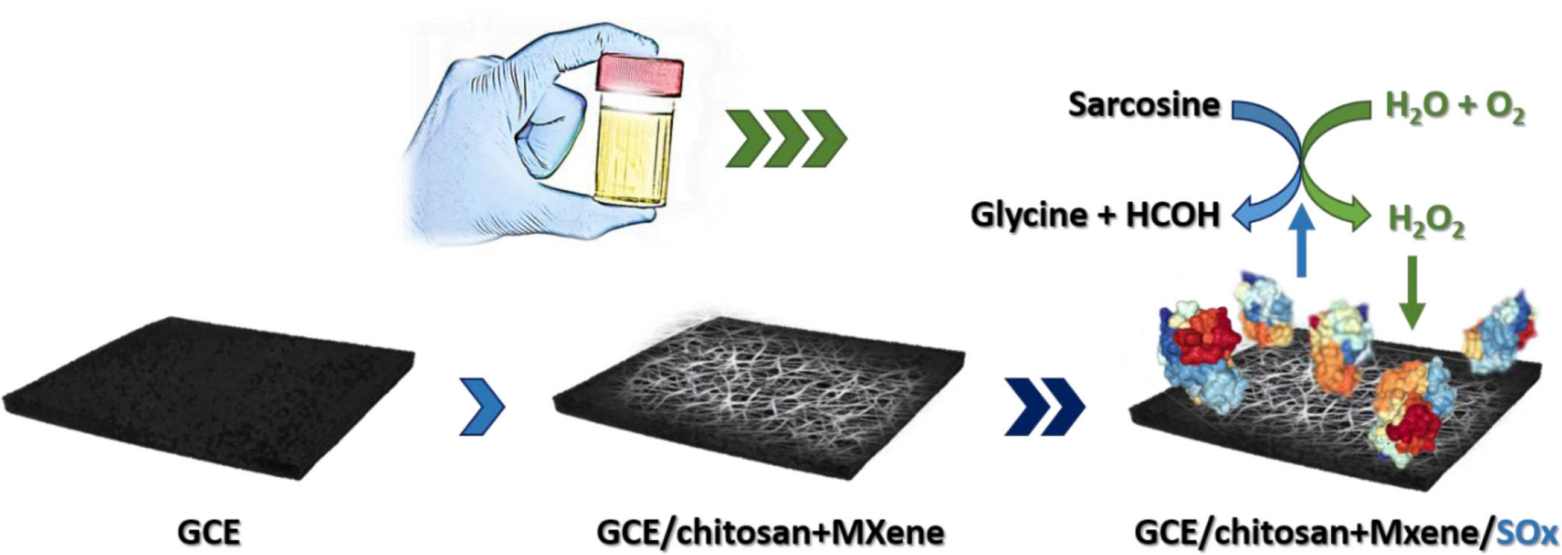
A graphical presentation of a glassy carbon electrode (GCE) modified using a MXene/chitosan nanocomposite as a support for sarcosine oxidase (SOx) immobilisation and indirect sarcosine detection in urine, based on hydrogen peroxide electrochemical reduction. SOx structure is adapted from the Protein Data Bank (code 1EL5) with permission from Reference [31]. Copyright (2000) American Chemical Society.

In this work, Ti_3_C_2_T_X_ (where T represents terminating functional groups such as =O, -OH or -F) MXene was used as an enzyme immobilisation support to construct a biosensor for sensitive detection of a small molecule—sarcosine (*N*-methylglycine, a by-product of glycine synthesis and degradation, Figure 1), a potential PCa biomarker [28, 29]. Sarcosine is also present in urine, which is a less complex matrix compared to human serum. Quantification of sarcosine was achieved indirectly via amperometric detection of H_2_O_2_ produced during the enzymatic reaction. A sensitive nanostructured MXene-based biosensor selectively detected sarcosine in urine in the concentration range of 0.02–5 µM, which is a clinically relevant range [30].

For the experimental section, see the Supplementary Materials.

## 2 Material and Methods

### 2.1 Materials

Sarcosine oxidase (SOx, enzyme commission number 1.5.3.1) from *Bacillus* *sp*. (lyophilised powder, 25–50 U mg^-1^), sarcosine (98%), glutaraldehyde solution (50 wt. % in H_2_O), chitosan (medium molecular weight), acetic acid, phosphate buffer (PB) components (KH_2_PO_4_ and K_2_HPO_4_) and Surine™ (a negative urine control for toxicology) were purchased from Sigma-Aldrich (St. Louis, MO, USA). Micropolish alumina powder (1.0 and 0.3 µm) and polishing pads for disc electrodes were purchased from Buehler (lake Bluff, IL, USA).

### 2.2 Preparation of MXene

MXene was prepared by in situ-formed hydrofluoric acid (HF) (reaction of lithium fluoride (LiF) with hydrochloric acid (HCl)) using a previously described protocol [32]. This MXene was previously shown to exhibit a good sensitivity towards H_2_O_2_ and some other small molecules as well.

### 2.3 Electrochemical Procedures

All electrochemical procedures were carried out on a laboratory potentiostat/galvanostat Autolab PGSTAT 302N with an impedimetric module (Ecochemie, Utrecht, Netherlands) with a GCE (*d* = 3 mm, Bioanalytical systems, West Lafayette, IN, USA) used as a working electrode. An Ag/AgCl/3 M KCl reference electrode and a counter platinum electrode (Bioanalytical systems, West Lafayette, IN, USA) were applied in a three-electrode cell system. Chronoamperometric detection of H_2_O_2_ (at different rpm) was performed on a rotating disc electrode employed as a working electrode (Metrohm Autolab, Ultrecht, Netherlands). Cyclic voltammetry and chronoamperometry were performed in 0.1 M phosphate buffer (PB) pH 7.4 purged with nitrogen for 15 min prior to use to eliminate the interference from an oxygen reduction reaction. Cyclic voltammetry measurements were run in the potential window from 0.1 V (volt) to -1.0 V at a scan rate of 100 mV s^-1^. Chronoamperometry measurements were run at a fixed potential value of -0.7 V. All electrochemical measurements were run under Nova Software 1.10 (Metrohm Autolab, Ultrecht, Netherlands), and data acquired were evaluated using OriginPro 9.1 (OriginLab, Northampton, MA, USA).

### 2.4 Construction of MXene-Based Sarcosine Biosensor

Prior to modification, a GCE with 3 mm diameter was polished on a polishing cloth with 1.0 and 0.3 µm alumina powder, then washed with distilled water and sonicated in ethanol and water successively. The MXene-based biosensor for determination of sarcosine was prepared by a simple drop-casting method. Firstly, 200 µL chitosan solution (Chi, 0.1% in 0.3% acetic acid) and 40 µL MXene dispersion (3 mg mL^-1^ in distilled water) were mixed and shaken overnight at a temperature of 20 °C and at a rotation speed of 1500 rpm. After preparation, 20 µL of the above-prepared mixture was cast onto the surface of polished GCEs. After drying, 10 µL of 5% or 1% glutaraldehyde solution was applied to the surface for 10 min. Construction and behaviour of the biosensor without using glutaraldehyde solution was also studied. Finally, 20 µL of the enzyme solution was cast onto the surface and allowed to dry at room temperature in a laminar box. Before electrochemical measurements, all the as-prepared electrodes were immersed in PB pH 7.4 to remove residual components. For the purpose of biosensor preparation and its performance, different configurations and nanocomposite preparation protocols were used (Supplementary Figure S1), i.e., (i) MXene+SOx (stock) mixed prior to drop-casting, (ii) MXene+SOx (10x diluted) mixed prior to drop-casting, (iii) layer-by-layer (LBL) drop-casting of MXene, SOx (10x diluted) and chitosan solution, (iv) MXene+SOx (10x diluted)+chitosan solution mixed prior to drop-casting, (v) all components mixed prior to drop-casting with SOx stock solution being desalted using Zeba Spin desalting columns (7k MWCO; Thermo, Waltham, MA, USA), alternatively MXene+chitosan mixed prior to drop-casting and subsequently activated using glutaraldehyde (GA) solutions (5%, 1% or no glutaraldehyde - GA) and incubated with desalted and diluted SOx. Among all these configurations, from the stability point of view (meaning reproducible performance, high sensitivity and no obvious dissolving of the prepared nanocomposite), only the configuration with mixed chitosan+MXene (overnight, 20 °C, 1500 rpm) prior to drop-casting, subsequently modified by LBL using desalted and diluted SOx with no GA activation, was chosen.

### 2.5 Contact Angle Measurements

Contact angle measurements were run on a portable instrument System E (Advex Instruments, Brno, Czech Republic) to obtain wetting angle and free surface energy for MXene-based interfaces. The droplet volume was 1 μL and the testing liquid was distilled water and diiodomethane. Free surface energy was determined using the two-liquid Owens–Wendt method, where the total surface energy *γ* consists of disperse *γ^d^* and polar *γ^p^* components. For each sample, the water and diiodomethane wetting angle was obtained as an average value of assays performed using 3 droplets.

### 2.6 Characterisation of MXene and MXene-Chitosan Composite

The morphology and structure of MXene and the as-prepared MXene-chitosan mixture were characterised by scanning electron microscopy (SEM) and atomic force microscopy (AFM). SEM images of MXene and the MXene-chitosan composite were recorded using a Nova NanoSEM 450 (FEI, Hillsboro, OR, USA) microscope, applying accelerating voltage. A peak force tapping mode atomic force microscopy (AFM, Scan Asyst, Bruker, Billerica, MA, USA) in air was carried out on a Bioscope Catalyst instrument and Olympus IX71 microscope in conjunction with NanoScope 8.15 software (Bruker, Billerica, MA, USA).

## 3 Results and Discussion

Five different configurations were tested during biosensor preparation (Supplementary Figure S1). Among all these configurations, from the stability point of view (meaning reproducible performance, high sensitivity and no obvious dissolving of the prepared nanocomposite), only the configuration with mixed chitosan+MXene (overnight, 20 °C, 1500 rpm) prior to drop-casting, subsequently modified by LBL using desalted and diluted SOx with no glutaraldehyde activation, was chosen. The main reason behind that decision was the fact that such biosensor offered the highest response, as shown below.

### 3.1 Characterisation of MXene

**Figure 2:**
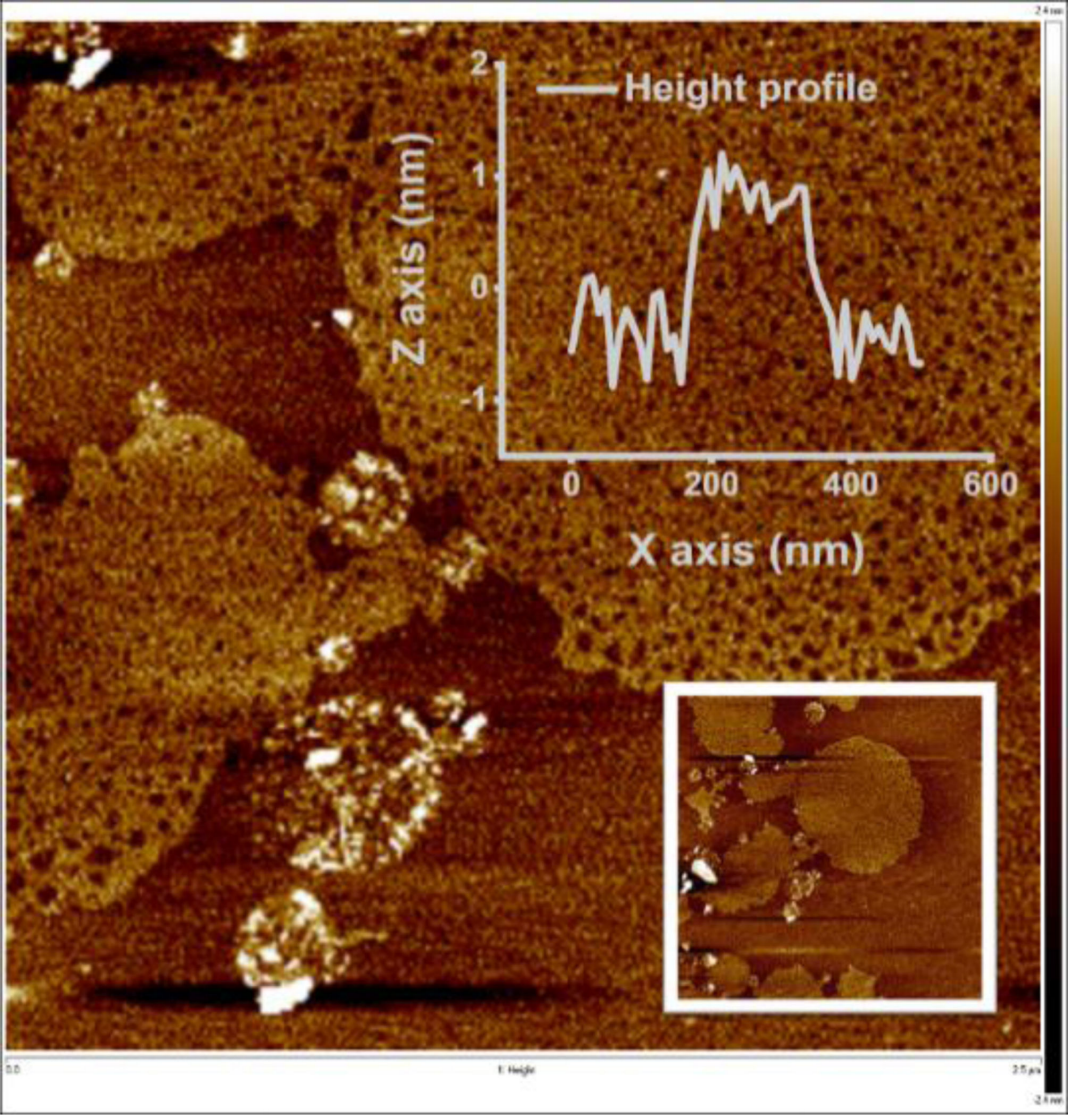
Atomic force microscopy (AFM) images showing individual MXene sheets (1 nm) observed in MXene solution after a week of storage in an aqueous solution, accompanied with a slight change in colour, most likely due to progressive oxidation and delamination. To prevent this from affecting the measurements, freshly prepared MXene solutions were used for every experiment.

Initial scanning electron microscopy-based characterisation of MXene showed the presence of MXene flakes with the size from a few μm to tens of μm (Supplementary Figure S2a–d). Atomic force microscopy showed the presence of MXene flakes with thickness of 20.2 ± 3.1 nm (Supplementary Figure S3c), which is in agreement with the literature [24, 27]. The thickness of the MXene-chitosan flake is shown in Supplementary Figure S3b. When MXene flakes were stored in water, after about a week, the MXene thickness decreased to 1 nm (Figure2).

Wetting angle and surface energy calculation using the Owens–Wendt method for two liquids (water and diiodomethane) were applied to further investigate the surface modifications during individual steps of biosensor preparation. The hydrophilicity of bare rotating glassy carbon electrode (GCE) (90.0 ± 2.3)° increased upon modification of the electrode by MXene (71.9 ±5.0)° and further by the MXene-chitosan composite (69.5 ± 3.6)°. After SOx adsorption, the biosensor surface becomes quite hydrophobic again (87.4 ± 4.9)° (Supplementary Figure S4).

### 3.2 Electrochemical Measurements

Since MXene undergoes irreversible anodic oxidation at +0.43 V [15, 16], cyclic voltammetry (CV) experiments were performed in a potential window from 0.1 V to -1.0 V in N_2_-purged PB pH 7.4. At this pH value, SOx has a maximum stability [33]. A direct comparison of the electrochemical activity towards 1.5 mM H_2_O_2_ on unmodified GCE and MXene-modified GCE is shown in Supplementary Figure S5, confirming significant redox activity of MXene-modified GCE towards H_2_O_2_ reduction. The effect of concentration of SOx applied for patterning of MXene-modified GCE was optimised in the range from 0.19 mg/mL to 0.77 mg/mL, with an optimal SOx concentration of 0.38 mg/mL identified.

**Figure 3:**
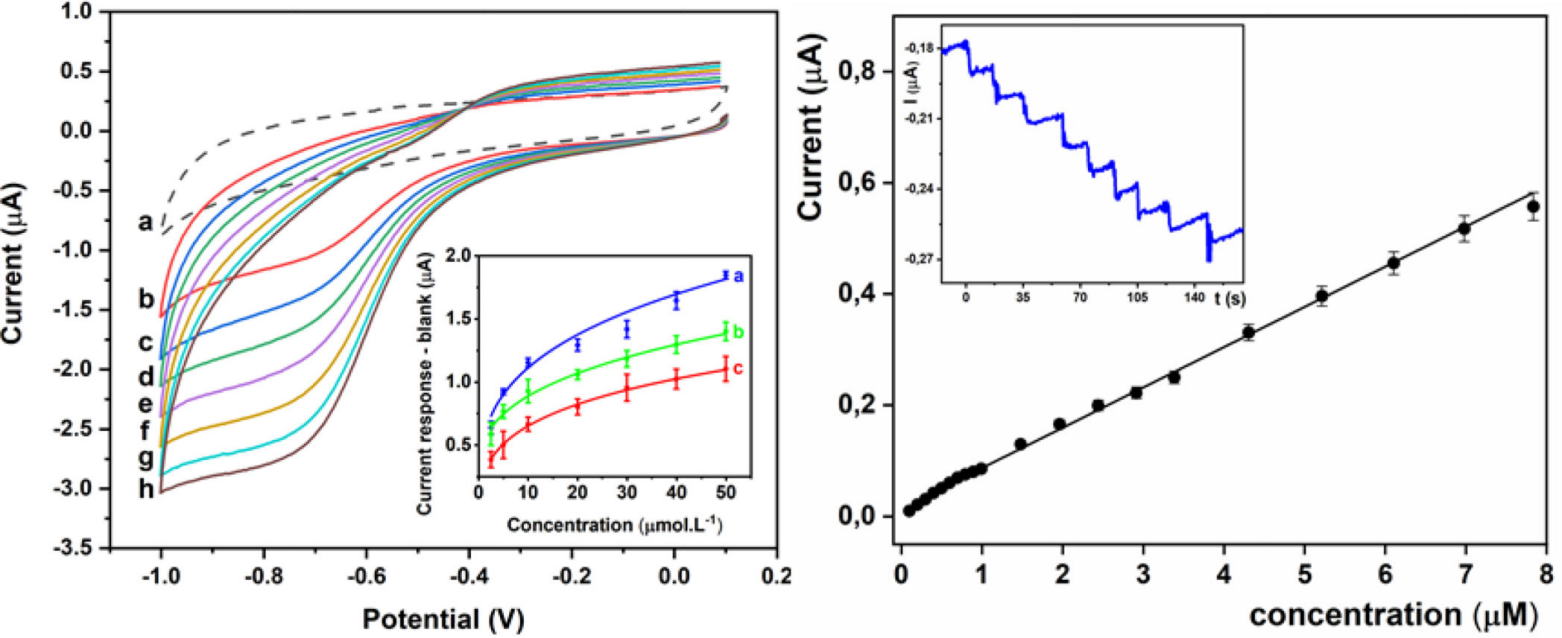
Left image: cyclic voltammograms CVs of the prepared SOx/MXene-Chi/GCE biosensor in 0.1 M PB pH 7.4 purged by N_2_ containing 0 (a), 2.5 (b), 5 (c), 10 (d), 20 (e), 30 (f), 40 (g), 50 (h) μM sarcosine at a scan rate of 100 mV s^-1^ in a potential window from -1.0 V to +0.1 V. Inset: Calibration plots obtained when using no glutaraldehyde (a, blue line), 1% glutaraldehyde (b, green line) and 5% glutaraldehyde (c, red line) during fabrication of the biosensor. Right image: Plot of current responses after subtraction of blank (current response obtained in the absence of sarcosine) of the SOx/MXene-Chi/GCE biosensor measured on a rotating disc glassy carbon electrode at -0.7 V and 900 rpm versus sarcosine concentration. Inset: a current versus time response of the SOx/MXene-Chi/GCE biosensor at -0.7 V to successive addition of stock sarcosine solution in stirred nitrogen-purged 0.1 M phosphate buffer, pH 7.4.

To evaluate the bioelectrochemical activity of the biosensor and to construct the corresponding calibration curves, the final sarcosine concentration was in the range of 2.5–50 µM (Figure 3, left). Electrochemical reduction of H_2_O_2_ produced by catalytic decomposition of sarcosine by SOx present on the surface of the electrode started at -0.4 V, with the peak maximum present at -0.7 V. The cathodic peak for H_2_O_2_ reduction increased with increasing biomarker concentration. Calibration curves were constructed by plotting current response versus sarcosine concentration in the first CV scan with subtraction of a background CV.

Immobilisation of SOx on the modified electrode was optimised using various glutaraldehyde concentrations (Figure 3, left inset). Fitting of calibration curves using a Hill model and Michaelis-Menten kinetics renders the following maximal current outputs (*I_max_*): (2.61 ± 0.15) μA (0% GA), (1.96 ± 0.26) μA (1% GA), and (1.42 ± 0.10) μA (5% GA). Thus, glutaraldehyde has a negative effect on the SOx activity, which can be explained by the formation of a multi-point attachment of SOx with the carrier, leading to the enzyme inactivation [34, 35]. Wang et al. [36] reported similar findings on deactivation of the enzyme by high concentrations of glutaraldehyde. One of the most important performance parameters of the biosensor is the limit of detection (LOD). The biosensor prepared without glutaraldehyde exhibits a limit of detection (LOD) (based on signal to noise ratio of 3) of 91.4 nM using CV. The biosensor devices constructed with the aid of glutaraldehyde exhibit a LOD of 79.9 nM (1% GA) or 110 nM (5% GA).

Chronoamperometry is a more sensitive electrochemical method compared to CV for indirect sarcosine detection. In order to obtain low LOD values, chronoamperometry on a rotating disc GCE at -0.7 V and 900 rpm was performed. Figure 3, right, shows a calibration plot of the SOx/MXene-Chi/GCE biosensor with a linear range up to 7.83 µM (R^2^=0.998, n=20) and LOD=18.0 nM (S/N=3). Moreover, the biosensor exhibits a very short response time of 2 s (Figure 3, right inset).

Our biosensor device belongs to the most sensitive MXene devices published so far, when considering LOD (Table 1). Additionally, our MXene-based sarcosine biosensor belongs to the most sensitive electrochemical biosensor devices for analysis of sarcosine, when comparing LOD (Table 2). For example the electrochemical SOx-based biosensor offered the following LOD: 430 nM for SOx immobilised on hollow magnetic Pt-Fe_3_O_4_@C nanospheres [37], 50 nM for organic transistor with grafted SOx on platinum-plated anodized aluminium oxide gate electrodes [30] and 10 nM for immobilisation of SOx nanoparticles onto gold electrode [38]. An optical-detection platform was also applied for preparation of SOx-biosensors with LOD down to a low μM range achieved for fused protein of SOx and fluorescent protein co-immobilised on silica particles with horseradish peroxidase [28, 39]. A hybrid type of the biosensor based on SOx immobilised on a non-structured gold electrode modified with CdSe/ZnS quantum dots offered a LOD of 0.11 mM, while detecting a photocurrent [40]. It is important to remember that the above-mentioned optical biosensor cannot be reused and requires sample pre-treatment. The performance of other SOx-based biosensors is summarised in a recent review paper [41]. Moreover, the MXene-biosensor developed here offers a really quick response towards the analyte, with a response time of 2 s, value equal to the best sarcosine biosensors [41].

### 3.3 Clinical Application of SOx/MXene-chi/GCE Biosensor

In order to evaluate potential application of the biosensor for analysis of real samples, the SOx/MXene-Chi/GCE device was applied for determination of sarcosine (0.1 to 0.5 µM) spiked into 10x diluted artificial urine (Surine™ negative urine control, Merck). The value of the obtained recovery index was 102.6%. This means that under such conditions, there were no interferents affecting the biosensor’s performance. The results prove that the as-fabricated biosensor is reliable tool for sarcosine detection in urine samples.

## 4 Conclusions

In this paper, a MXene-based biosensing platform was successfully fabricated for indirect sensitive detection of sarcosine. The sarcosine biosensor displayed low detection limit of 18 nM and a linear range up to 7.8 µM for sarcosine, with correlation coefficient of 0.998 and a response time of 2 s. The SOx/MXene-Chi/GCE device was further used for sarcosine determination in artificial urine with a recovery index (defined as the concentration of the analyte measured by the assay divided by concentration of sarcosine spiked into artificial urine and expressed in %) of 102.6%. These excellent operational parameters make MXene a potential electrochemical biosensing platform for protein-based biosensors with application in biomedical detection and diagnostics. In future work, we would like to test the device for analysis of human urine samples from healthy individuals and patients with prostate cancer.

## Supplementary Material

Figs. S1-S5

Supplementary Materials

## Figures and Tables

**Table 1:** Electrochemical biosensors based on MXene with integration of redox proteins/enzymes.

Enzyme/protein	MXene patterning	Immobilization	Analyte	LOD (nM)	Reference
haemoglobin (Hb)	MXene	Hb glued *via* Nf	H2O_2_	20	[23]
haemoglobin (Hb)	MXene	Hb glued *via* Nf	NO_2_^-^	120	[24]
haemoglobin (Hb)	TiO_2_ on MXene	Hb glued *via* Nf	H2O_2_	14	[22]
tyrosinase (Tyr)	MXene	Tyr glued *via* Chi	phenol	12	[27]
glucose oxidase	AuNPs on MXene	GOx adsorbed on Nf-AuNP/MXene	glucose	5900	[25]
SOx	MXene	SOx glued *via* Chi	sarcosine	18	This work

Abbreviations: LOD—limit of detection; Nf—nafion; Chi—chitosan; NPs—nanoparticles.

**Table 2:** Analytical performance of SOx-based electrochemical biosensors.

Interface	LOD (nM)	Working range (nM)	Reference
platinum-plated anodised aluminium oxide electrode	50	50–100,000	[30]
Hybrid: Pt nanoparticles with hollow Fe3O4 nanospheres	430	500–60,000	[37]
Modified SPCE	16	10–100	[42]
SOxNPs/AuE	10	100–100,000	[38]
SOx/Pt–Fe_3_O_4_@C nanocomposite/GCE	430	500–60,000	[37]
Pt-supported organic/inorganic hybrid mesoporous NPs	130	1000–70,000	[43]
Riboflavin/AuPt-PPy/graphene-chitosan-modified GCE	680	2500–600,000	[44]
nanoPt@porous zeolitic imidazolate framework-8	1060	5000–30,000	[45]
SOx/MXene-Chi/GCE	18	36–7800	This work

Abbreviations: SPCE – screen printed carbon electrode; PPy – polypyrrole; AuE – gold electrode; Chi - chitosan
