# Supplementary material for: Ultrasensitive Ti3C2TX MXene/Chitosan Nanocomposite-Based Amperometric Biosensor for Detection of Potential Prostate Cancer Marker in Urine Samples
Source: Processes (Basel). Author manuscript; Available in PMC 2020 Dec 9. (PMC7116456; doi:10.3390/pr8050580)
Supplement: Figs. S1-S5 [file EMS106554-supplement-Figs__S1_S5.docx]

**SUPPORTING INFORMATION**

Ultrasensitive Ti_3_C_2_T_X_ MXene/Chitosan Nanocomposite-Based Amperometric Biosensor for Detection of Potential Prostate Cancer Marker in Urine Samples

Stefania Hroncekova ^1^, Tomas Bertok ^1^, Michal Hires ^1^, Eduard Jane ^1^, Lenka Lorencova ^1^, Alica Vikartovska ^1^, Aisha Tanvir ^2^, Peter Kasak ^2^ and Jan Tkac^1,^*

^1^ Institute of Chemistry, Slovak Academy of Sciences, Dubravska cesta 9, Bratislava 845 38, Slovak Republic; [stefania.hroncekova@savba.sk](javascript:void(0);) (S.H.), [tomas.bertok@savba.sk](javascript:void(0);) (T.B.), [michal.hires@savba.sk](javascript:void(0);) (M.H.), [eduard.jane@savba.sk](javascript:void(0);) (E.J.), [lenka.lorencova@savba.sk](javascript:void(0);) (L.L.), [alica.vikartovska@savba.sk](javascript:void(0);) (A.V.)

^2^ Center for Advanced Materials, Qatar University, P. O. BOX 2713, Doha, Qatar; [atanvir@qu.edu.qa](javascript:void(0);) (A.T.), [peter.kasak@qu.edu.qa](javascript:void(0);) (P.K.)

***** Correspondence: [jan.tkac@savba.sk](mailto:jan.tkac@savba.sk)


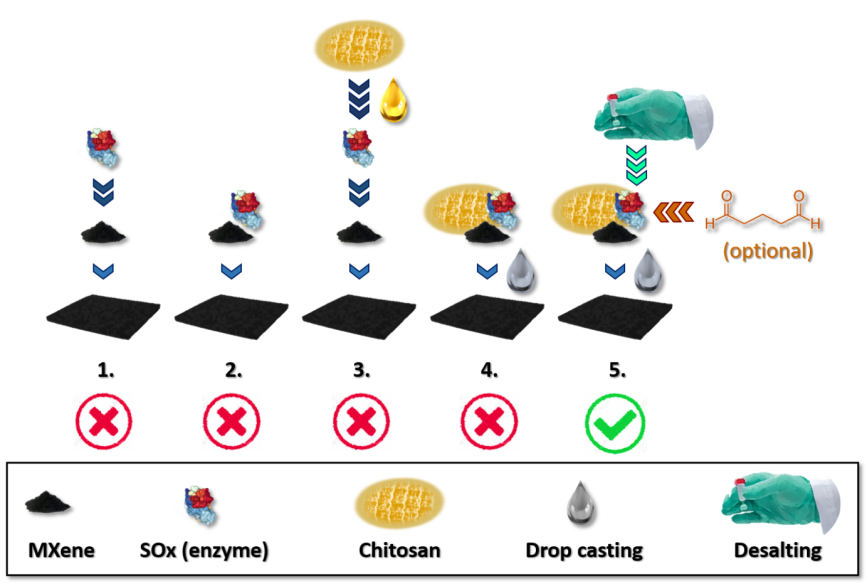


**Figure S1:** Graphical presentation of different configurations of sarcosine biosensor investigated in this study: 1. GCE modified with MXene solution (drop casting method) and subsequently with SOx enzyme, 2. GCE modified with MXene and SOx enzyme mixed prior to drop casting - in a single step, 3. layer-by-layer modification of GCE, with MXene as a support, SOx enzyme and finally with chitosan to increase the stability, 4. all three components mixed prior to drop casting, 5. all three component mixed prior to drop casting, with SOx enzyme being desalted and with (optional) glutaraldehyde crosslinking to increase the stability even further. Only this last configuration was stable enough to perform repeated measurements in aqueous solutions.

1. **Results and discussion**
   1. **Microscopic characterization of MXene and MXene-chitosan composite**

Scanning electron microscopy (SEM) analysis was used to observe the microstructures of the samples. SEM images revealed aggregation of MXene flakes differing in size. Size of few µm (**Fig. S2a**) or size with more than 10 µm was observed (**Fig. S2b**). The results obtained are in a good agreement with results obtained in a previous study [2]. **Fig. S2a** shows that prepared MXene dispersion contains microscale-sized sheets with a multilayer nanostructure. Formation of various sized MXene-chitosan composite aggregates with porous structure was also detected (**Fig. S2c-d**). 2D multilayered porous nanostructure of MXene-based layers provides great surface area for SOx immobilization. This leads to improved performance and sensitivity of fabricated biosensor.


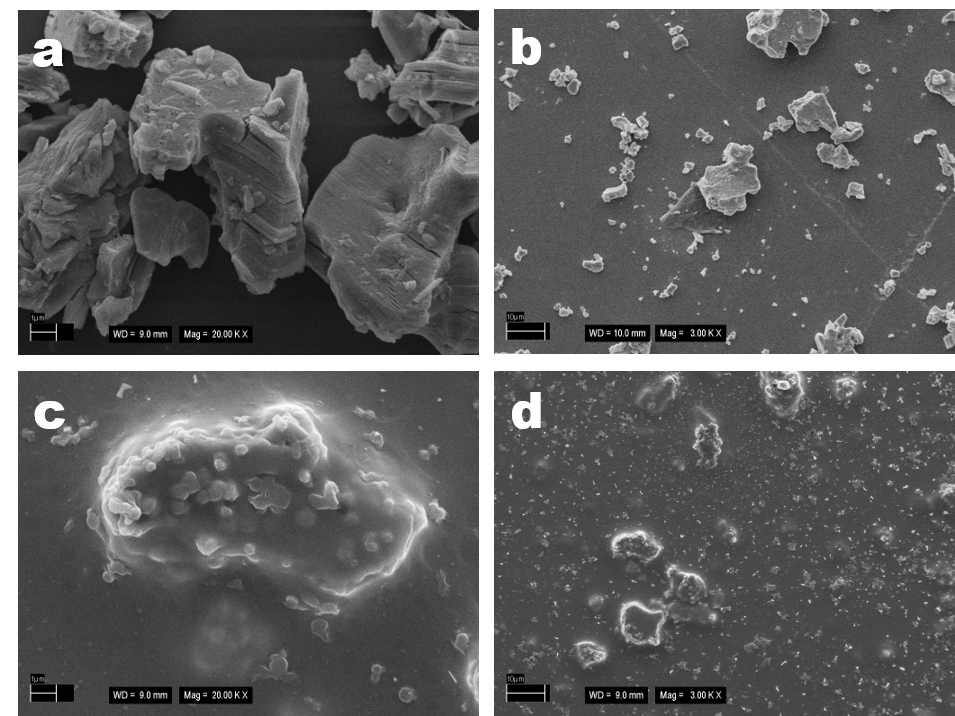


**Figure S2:** SEM images showing (**a**) an unmodified Ti_3_C_2_T_X_ MXene flakes, 20k magnification (**b**), same unmodified flakes, 3k magnification and (**c**) Ti_3_C_2_T_X_ MXene/chitosan nanocomposite using 20k and (**d**) 3k magnification. Individual MXene sheets enwrapped in the chitosan is clearly visible.

- 1. **Atomic force microscopy (AFM)**

The main aim for performing AFM measurements was to investigate and see the surface morphology of prepared MXene (**Fig. S3a**) and of a MXene-chitosan composite (**Fig. S3b**) layers before enzyme immobilization took place. Height profile analysis results revealed increase in MXene-chitosan layer height compared to MXene. MXene-chitosan flakes were about 10 times thicker than those consisting of MXene (**Fig. S3a, S3b**). Average thickness of one MXene nanosheet was calculated to be (20.2 ± 3.1) nm (**Fig. S3c**). Achieved value is similar to results published so far [3, 4]. 1 nm thick MXene flakes (together with a slight change in the solution colour) could be observed after about a week of MXene storage in water, suggesting possible oxidation of MXene, which was further prevented by de-aeration using N_2_ or Ar and preparing fresh MXene solution *prior* to each experiment.


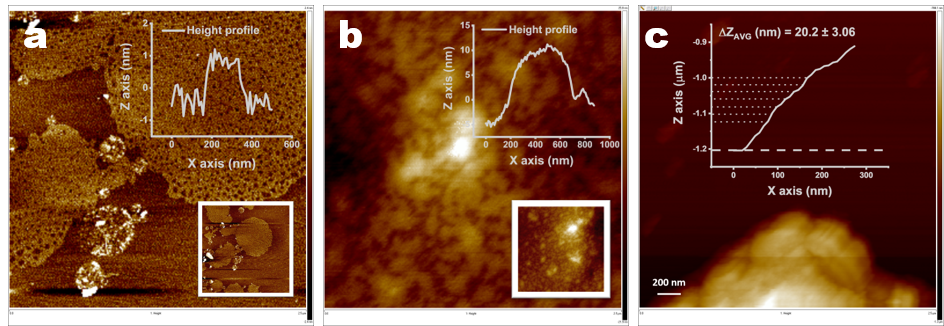


**Figure S3:** AFM images showing (**a**) individual MXene sheets (~1 nm) observed in MXene solution after a week of storage in an aqueous solution, accompanied with a slight change in colour, (**b**) individual MXene flake enwrapped in chitosan and (**c**) the edge of an unmodified (bare) MXene, where we were able to calculate the thickness of a separated nanosheet as ~20.1 nm – a value in a good correlation with literature.


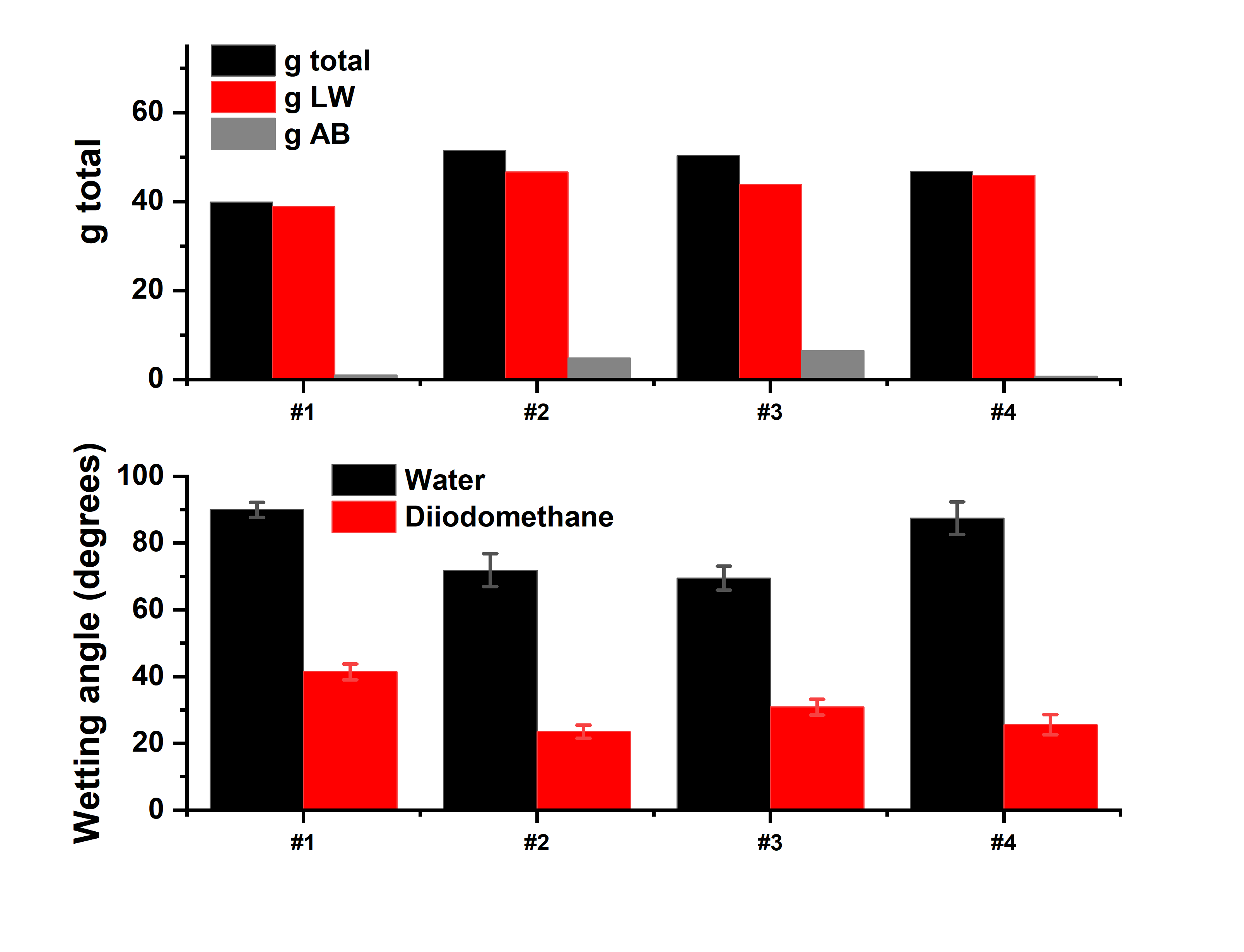


**Figure S4:** Graphical presentation of free surface energy (upper row) and wetting angle measurements (lower row) for #1: bare GCE, #2: MXene-modified GCE, #3: MXene/chitosan-modified GCE and #4: SOx-MXene/chitosan-modified GCE. Owen-Wendt model for two liquids (water and diiodomethane) was used.





**Figure S5:** Representative blank-subtracted CV scans run at GCE and GCE/MXene in 1.5 mM H_2_O_2_ in 0.1 M PB pH 7.0.

1. **References**

1. Alhabeb, M.; Maleski, K.; Anasori, B.; Lelyukh, P.; Clark, L.; Sin, S.; Gogotsi, Y., Guidelines for Synthesis and Processing of Two-Dimensional Titanium Carbide (Ti3C2Tx MXene). *Chem. Mater.* **2017,** 29, (18), 7633-7644.

2. Lorencova, L.; Bertok, T.; Dosekova, E.; Holazova, A.; Paprckova, D.; Vikartovska, A.; Sasinkova, V.; Filip, J.; Kasak, P.; Jerigova, M.; Velic, D.; Mahmoud, K. A.; Tkac, J., Electrochemical performance of Ti3C2Tx MXene in aqueous media: towards ultrasensitive H2O2 sensing. *Electrochim. Acta* **2017,** 235, 471-479.

3. Wu, L.; Lu, X.; Dhanjai; Wu, Z. S.; Dong, Y.; Wang, X.; Zheng, S.; Chen, J., 2D transition metal carbide MXene as a robust biosensing platform for enzyme immobilization and ultrasensitive detection of phenol. *Biosens. Bioelectron.* **2018,** 107, 69-75.

4. Liu, H.; Duan, C.; Yang, C.; Shen, W.; Wang, F.; Zhu, Z., A novel nitrite biosensor based on the direct electrochemistry of hemoglobin immobilized on MXene-Ti3C2. *Sens. Actuat. B: Chem.* **2015,** 218, 60-66.
