# Supplementary material for: Ultrasensitive Ti3C2TX MXene/Chitosan Nanocomposite-Based Amperometric Biosensor for Detection of Potential Prostate Cancer Marker in Urine Samples
Source: Processes (Basel). Author manuscript; Available in PMC 2020 Dec 9. (PMC7116456; doi:10.3390/pr8050580)
Supplement: Supplementary Materials [file EMS106554-supplement-Supplementary_Materials.docx]

**Supplementary Materials:** The following are available online at www.mdpi.com/xxx/s1; Figure S1: Graphical presentation of different configurations of sarcosine biosensor investigated in this study: 1. GCE modified with MXene solution (drop casting method) and subsequently with SOx enzyme, 2. GCE modified with MXene and SOx enzyme mixed prior to drop casting—in a single step, 3. layer-by-layer modification of GCE, with MXene as a support, SOx enzyme, and finally, with chitosan to increase the stability, 4. all three components mixed prior to drop casting, 5. all three components mixed prior to drop casting, with SOx enzyme being desalted and with (optional) glutaraldehyde crosslinking to increase the stability even further. Only this last configuration was stable enough to perform repeated measurements in aqueous solutions. Figure S2: SEM images showing (**a**) unmodified Ti_3_C_2_T_X_ MXene flakes, 20k magnification, (**b**) the same unmodified flakes, 3k magnification, and (**c**) Ti_3_C_2_T_X_ MXene/chitosan nanocomposite using 20k and (**d**) 3k magnification. Individual MXene sheets enwrapped in the chitosan are clearly visible. Figure S3: AFM images showing (**a**) individual MXene sheets (~1 nm) observed in MXene solution after a week of storage in an aqueous solution, accompanied with a slight change in colour, (**b**) individual MXene flake enwrapped in chitosan, and (**c**) the edge of an unmodified (bare) MXene, where we were able to calculate the thickness of a separated nanosheet as ~20.1 nm—a value in good correlation with the literature. Figure S4: Graphical presentation of free surface energy (upper row) and wetting angle measurements (lower row) for #1: bare GCE, #2: MXene-modified GCE, #3: MXene/chitosan-modified GCE and #4: SOx-MXene/chitosan-modified GCE. The Owen–Wendt model for two liquids (water and diiodomethane) was used. Figure S5: Representative blank-subtracted CV scans run at GCE and GCE/MXene in 1.5 mM H_2_O_2_ in 0.1 M PB pH 7.0.
